# PeakForest: a multi-platform digital infrastructure for interoperable metabolite spectral data and metadata management

**DOI:** 10.1007/s11306-022-01899-3

**Published:** 2022-06-14

**Authors:** Nils Paulhe, Cécile Canlet, Annelaure Damont, Lindsay Peyriga, Stéphanie Durand, Catherine Deborde, Sandra Alves, Stephane Bernillon, Thierry Berton, Raphael Bir, Alyssa Bouville, Edern Cahoreau, Delphine Centeno, Robin Costantino, Laurent Debrauwer, Alexis Delabrière, Christophe Duperier, Sylvain Emery, Amelie Flandin, Ulli Hohenester, Daniel Jacob, Charlotte Joly, Cyril Jousse, Marie Lagree, Nadia Lamari, Marie Lefebvre, Claire Lopez-Piffet, Bernard Lyan, Mickael Maucourt, Carole Migne, Marie-Francoise Olivier, Estelle Rathahao-Paris, Pierre Petriacq, Julie Pinelli, Léa Roch, Pierrick Roger, Simon Roques, Jean-Claude Tabet, Marie Tremblay-Franco, Mounir Traïkia, Anna Warnet, Vanessa Zhendre, Dominique Rolin, Fabien Jourdan, Etienne Thévenot, Annick Moing, Emilien Jamin, François Fenaille, Christophe Junot, Estelle Pujos-Guillot, Franck Giacomoni

**Affiliations:** 1 Université Clermont Auvergne, INRAE, UNH, Plateforme d’Exploration du Métabolisme, MetaboHUB Clermont, Clermont-Ferrand, France; 2 Toxalim (Research Center in Food Toxicology), Université de Toulouse, INRAE, ENVT, INP-Purpan, UPS, MetaboHUB, 31300 Toulouse, France; 3 Département Médicaments et Technologies pour la Santé (DMTS), Université Paris-Saclay, CEA, INRAE, MetaboHUB, 91191 Gif sur Yvette, France; 4 MetaboHUB-MetaToul, National Infrastructure of Metabolomics & Fluxomics (ANR-11-INBS-0010), 31077 Toulouse, France; 5 Université de Bordeaux, INRAE, Biologie du Fruit et Pathologie, UMR 1332, Bordeaux Metabolome, MetaboHUB, PHENOME-EMPHASIS, 71 av E. Bourlaux, 33140 Villenave d’Ornon, France

**Keywords:** Curation, Database, FAIR, Interoperability, Metabolite identification, Spectral library

## Abstract

**Introduction:**

Accuracy of feature annotation and metabolite identification in biological samples is a key element in metabolomics research. However, the annotation process is often hampered by the lack of spectral reference data in experimental conditions, as well as logistical difficulties in the spectral data management and exchange of annotations between laboratories.

**Objectives:**

To design an open-source infrastructure allowing hosting both nuclear magnetic resonance (NMR) and mass spectra (MS), with an ergonomic Web interface and Web services to support metabolite annotation and laboratory data management.

**Methods:**

We developed the PeakForest infrastructure, an open-source Java tool with automatic programming interfaces that can be deployed locally to organize spectral data for metabolome annotation in laboratories. Standardized operating procedures and formats were included to ensure data quality and interoperability, in line with international recommendations and FAIR principles.

**Results:**

PeakForest is able to capture and store experimental spectral MS and NMR metadata as well as collect and display signal annotations. This modular system provides a structured database with inbuilt tools to curate information, browse and reuse spectral information in data treatment. PeakForest offers data formalization and centralization at the laboratory level, facilitating shared spectral data across laboratories and integration into public databases.

**Conclusion:**

PeakForest is a comprehensive resource which addresses a technical bottleneck, namely large-scale spectral data annotation and metabolite identification for metabolomics laboratories with multiple instruments. PeakForest databases can be used in conjunction with bespoke data analysis pipelines in the Galaxy environment, offering the opportunity to meet the evolving needs of metabolomics research. Developed and tested by the French metabolomics community, PeakForest is freely-available at https://github.com/peakforest.

**Supplementary Information:**

The online version contains supplementary material available at 10.1007/s11306-022-01899-3.

## Introduction

Over the last 20 years, untargeted metabolomics has developed into a powerful phenotyping tool to better understand biological systems and identify associated biomarkers. This approach, based on multiple analytical platforms, generates massive and complex data that need appropriate analyses to extract biologically-meaningful information (Alonso et al., 2015). In particular, downstream analysis of metabolomics data requires annotation and identification of features in metabolic profiles. In order to move towards standardized identification methods, the metabolomics community has proposed a definition of metabolite identification accuracy ranges from unknown compounds to confidently-identified compounds (Sumner et al., 2007). This classification undergoes regular amendments, led by the Metabolomics Society and international consortia (Creek et al., 2014; Malinowska & Viant, 2019), in order to reduce ambiguities, better account for chemical structures and improve metabolite annotation confidence. However, metabolite annotation remains a major bottleneck in untargeted mass spectrometry (MS) and nuclear magnetic resonance (NMR) metabolomics (Dona et al., 2016; Nash & Dunn, 2019), and the development of workflows and dedicated tools is critical for accurate metabolite identification (Misra, 2021).

In high-resolution MS or NMR datasets, the first annotation step generally consists of matching experimental accurate masses or chemical shifts with those contained in public and commercial databases. Vendors and associated companies offer a large number of solutions to mine MS or NMR spectral libraries directly from their own instruments (e.g. NIST™) and proprietary databases such as Chenomx™. However, these solutions can lack interoperability with academic bioinformatic tools and often require specific acquisition conditions that oblige operators to adopt operating procedures that differ from experimental conditions. Public libraries have the advantage of hosting large-scale data from different organisms and technical instruments, and include a multiplicity of cross-references concerning biological or chemical information (Vinaixa et al., 2016). Valuable metabolomics resources in the field include MS libraries such as Wishart laboratory databases (Wishart et al., 2018), the Global Natural Product Social Molecular Networking (GNPS, Wang et al., 2016), LIPID MAPS (Fahy et al., 2009), MassBank (Horai et al., 2010), Metlin (Guijas et al., 2018), MoNA or mzCloud™. The NMR community shares resources with MS, such as the human metabolome database (Wishart et al., 2009), and NMR-specific banks such as the BioMagResBank (Ulrich et al., 2007), the Birmingham Metabolite Library (Ludwig et al., 2012), and more generalist banks including NMRShiftDB (Kuhn & Schlörer, 2015) for organic compounds. This relative abundancy of resources represents a large data heterogeneity, from “in silico” spectra derived from modeling and/or references to highly-curated spectra obtained from pure compounds, and remains far from containing experimental data of all known metabolites (Vinaixa et al., 2016). As with commercial resources, the exchange and interoperability of annotations from one laboratory to another can be difficult due to different formatting requirements. Moreover, existing databases are not always easy to increment with new compounds or spectra from external users due to logistical constraints or lack of recommendations (Johnson & Lange, 2015; Spicer et al., 2017).

Database interoperability simplifies the mining of multiple databases, promotes efficient use of metabolomics data and is at the heart of FAIR guidelines (“Findable, Accessible, Interoperable, Reusable”). At the metadata level, common vocabulary and consensual description levels in data collecting steps are required (Alseekh et al., 2021). At the computing level, common formats and application programming interfaces (API) are needed to enable data exchange between databases and connect databases to data treatment tools (Anwar et al., 2021; Merlet et al., 2016). Recent calls in the metabolomics community emphasize the need to develop adapted informatics infrastructures for laboratories based on FAIR principles in order to improve the exchange and interoperability of annotations from one laboratory to another and the sharing (and inclusion) of local spectral libraries with reference databases (Haug et al., 2017; Sansone et al., 2012). However, laboratory-based systems can be limited in their capacity to centralize all the descriptive and analytical characteristics of reference compounds, and may face difficulties in exporting spectral data in recommended formats such as mzML (Martens et al., 2011) or nmrML (Schober et al., 2018).

In this paper we present PeakForest, an open-source and open access infrastructure which hosts for the first time both NMR and mass spectra, with an ergonomic Web interface and Web services to support metabolite annotation and laboratory data management. This resource, deployable locally, facilitates the production of high-quality spectral records and their submission to international repositories such as MassBank Europe^1^ or MassBank of North America^2^ (MoNA). Building on the expertise and the experience of members of the French national metabolomics and fluxomics infrastructure (MetaboHUB), PeakForest integrates database interoperability at the metadata- and the computing-level, in order to further the implementation of FAIR spectral databases within the metabolomics community.

## Materials and methods

**Fig. 1:**
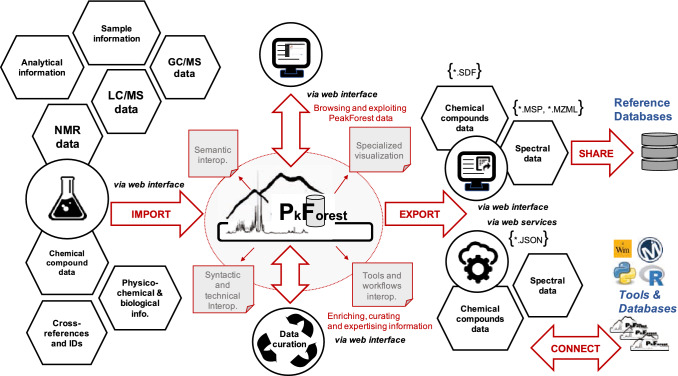
PeakForest database inputs and outputs

PeakForest is a modular framework including a database, a graphical user interface and a Web API. It is designed to ensure interoperability, easy deployment and code sustainability. Particular care has been taken to ensure resource security and intellectual property (data author, licensing, bibliographic references). PeakForest manages metabolomic data during the metabolite identification process, from the acquisition of chemical standard spectra, the annotation of spectra in biological matrices via external peak matching tools, to the spectra linkage with external resources for biological interpretation or publication (PeakForest has been specifically designed to be easily interfaced with external tools). Users can import compounds and spectral data manually extracted from raw data, curate information and exploit this knowledge via data export or PeakForest Web services connected tools (Fig. 1). PeakForest stores metadata related to raw data rather than raw data itself to minimize storage space usage.

### PeakForest framework technical specifications

PeakForest is a Java Web application (version 8) and has been designed as an API’s toolbox (Online Resource 1). The architecture leverages a complete data model, integrating entities as chemical compounds and spectra.

PeakForest proposes representational state transfer (*REST*) protocol-based Web services allowing programmatic data access to external resources and tools. Based on the OpenApi (v3.0) standard, any developer can generate a PeakForest Web services client in common programming languages (Python, R…). Centralizing the REST specifications in a unique OpenAPI compliant file allows consistent documentation, up-to-date code and exchanges between servers and clients.

All PeakForest components use the same Java APIs. The Web application and the REST documentation are hosted on a Tomcat server (version 7) allowing the application to be run using a basic Java virtual machine (version 8). Full details and technical aspects are described in the official install documentation^3^ and short tutorials.^4^ In order to facilitate the deployment of local PeakForest databases within laboratories, users can be attributed different privileges and permissions in the PeakForest system (Online Resource 1). PeakForest is available on DockerHub,^5^ making possible the run of several specialized databases on a unique server.

### Source and code project

PeakForest is a free and open-source project under the CECILL-2.1^6^ license, published on GitHub.^7^ Issues and incidents can be reported on this PeakForest official public repository. The PeakForest database model and project code have been deposited on APP,^8^ a European organization for the protection of authors and publishers of digital creations (ID: *IDDN.FR.001.180009.000.S.C.2021.000.31230*).

## Results

PeakForest manages metabolomics data including chemical compound descriptors and different types of analytical spectra. Main Web interface functionalities are detailed below:

### Building a PeakForest database

#### Compounds data and metadata inputs

**Fig. 2:**
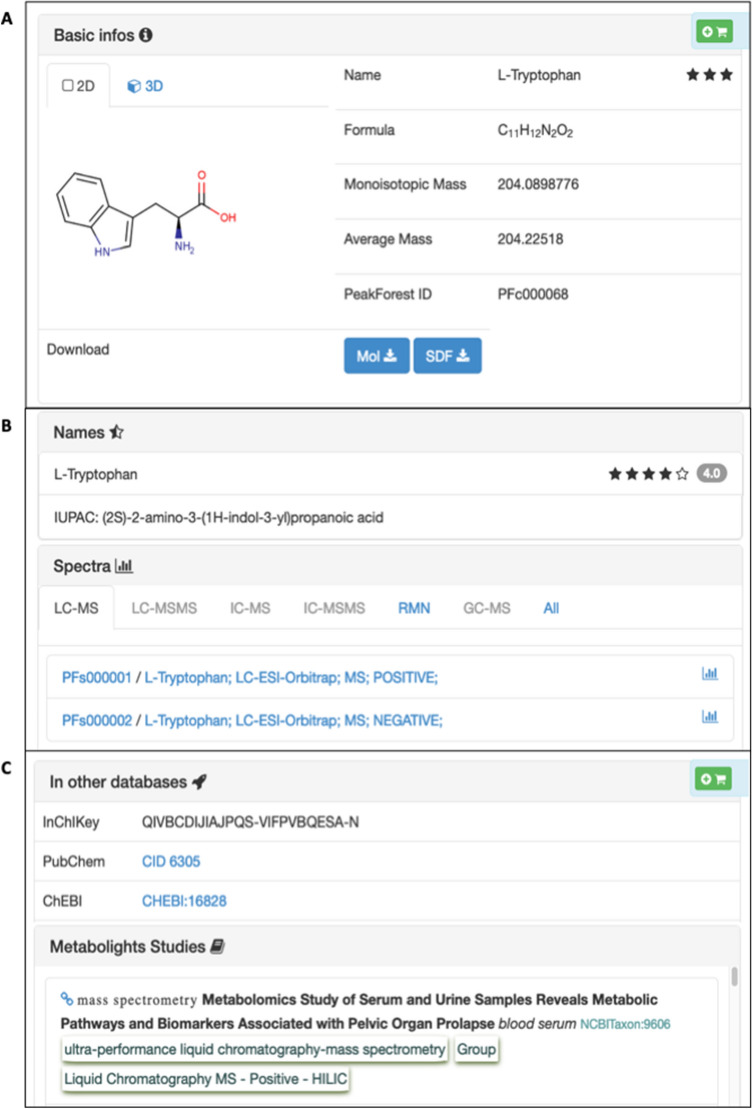
Example of a chemical compound card (L-tryptophan) with compound basic information (**A**), related names and spectra parts (**B**) and externals identifiers and related Metabolights studies (**C**)

Adapted for compounds already present in public databases, the import module allows the addition of individual chemical compounds, attributes a specific internal identifier, and creates the associated compound card from an InChIKey or a common name (Fig. 2). An “addition assistant” checks if the compound is already present in the database, and proposes compound candidates based on the Fiehn ‘Chemical Translation Service’ (Wohlgemuth et al., 2010) and the PubChem PUG-REST service (Kim et al., 2018) where necessary to complete the missing lnChIKey data. Using the same Web services, this module fills missing information such as synonyms, structural representation of compounds and provides external cross-references where available. The module computes accurate mass, formulae and SMILES (simplified molecular-input line-entry system) as well as running molecule structure image depiction with *OpenBabel*.^9^ A batch system is also available to import a large compound list (Online Resource 2) and create associated compound cards based on minimal compound information found in the imported file (common name, InChI and InChIKey are mandatory for this function to operate). Use of InChIKey identifiers avoids difficulties associated with ambiguous compound names.

Each compound card is organized in different sections. The first section presents general compound information (common name, synonyms, molecular formula, accurate and average masses). The second section is related to structural data with common numerical molecule representations in MDL Molfile (Dalby et al., 1992), Canonical SMILES (O’Boyle, 2012), InChI and InChIKey formats (Goodman et al., 2021; Southan, 2013) and *2D* and *3D* molecule images. A “cross-reference identifiers” section includes four selected external chemical compounds bank references, with ChEBI (Hastings et al., 2016), PubChem (Kim et al., 2021), KEGG (Kanehisa et al., 2016), and HMDB (Wishart et al., 2018) and a modular system to add any Web hyperlinks from specific biological knowledge banks or metabolic networks databases. The compound card also gives direct access to the full list of spectral cards available on the database instance, grouped by analytical techniques.

#### Spectral data and metadata inputs

The spectral module allows the addition of new spectra to the local PeakForest database. PeakForest supports a large range of spectral types, in line with common metabolomics analytical technologies: NMR-1D (^1^H, ^13^C), NMR-2D (JRES, COSY, TOCSY, NOESY, HSQC, HMBC), LC–MS(/MS^n^), FIA-MS(/MS^n^) and GC–MS. During the spectral import process, all spectra are associated with a chemical compound, and qualified as either a chemical standard, part of a chemical standard-mix, present in a reference biological matrix (e.g. NIST plasma) or in a biological (or environmental) matrix. Spectral data models have been designed based on expert advice using standardized metadata describing NMR and MS acquisition methods. Heterogeneity of analytical instruments is considered, and unique descriptors^10^ are proposed to enable metadata sharing within the metabolomics community. PeakForest uses IUPAC nomenclature and MassBank consortium proposal^11^ for mass spectrometry (Murray et al., 2013) and suggests the use of standardized and chemically-consistent ion annotation procedure following the recommendations of Damont et al., 2019 for user-defined peak attributions (Damont et al., 2019). The database structure also supports information on sample preparation and species origins.

**Fig. 3:**
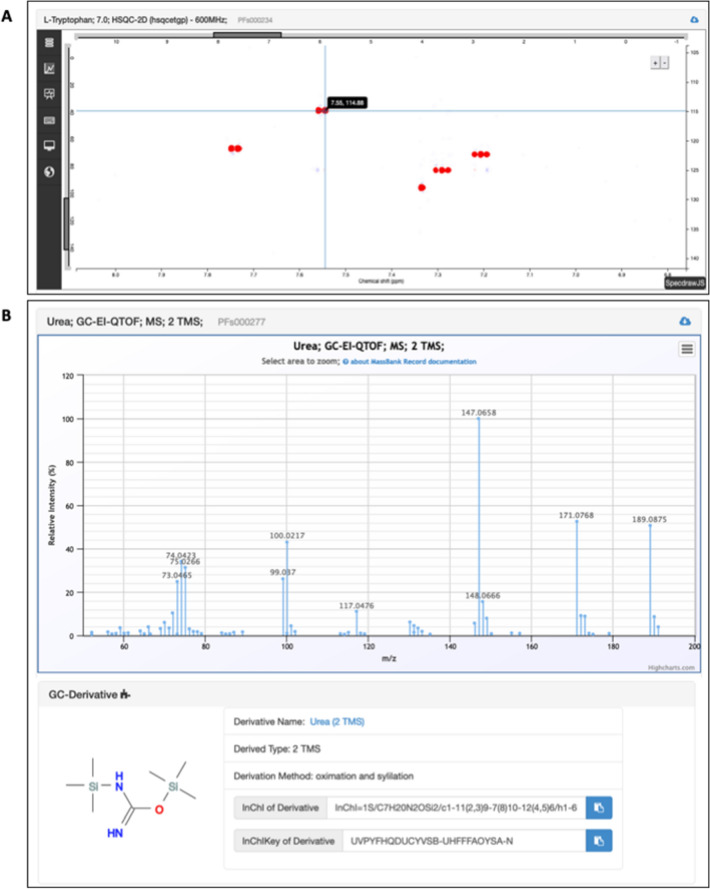
Spectral card examples related to L-Tryptophan; pH 7.0; HSQC-2D (hsqcetgp)—600 MHz card (**A**) and Urea; GC-EI-QTOF; MS; 2 TMS card (**B**)

An interactive and adaptive Web form is provided for spectrum addition, with four major steps for a LC–MS spectral import example: spectrum type, sample type, liquid chromatography condition and MS analyser information. An excel-like file template can also be generated with prefilled and predefined data and metadata based on user analytical methods (example templates in Online Resources 3–6). A batch system exists for large-scale imports of new spectra. All imported spectra generate spectrum cards with a short description of sample preparation (centrifugation, purification, dilution or derivatization conditions). Layout of spectrum cards depends on spectral type (Table 1, Fig. 3). Each spectrum is provided with a specific internal identifier and a splash identifier is also computed for LC–MS data (Wohlgemuth et al., 2016). The summary of compounds and spectral data origins is available in Online Resource 7.

**Table 1:** Spectral data and metadata group view with links of corresponding templates (available in the supplementary material section) and potential usage for users

Metadata group label	Context and template part link	Metadata usage
*Sample metadata*	All spectra templates “sample” sheet	Informs users about the spectrum’s type (single chemical compound, mix of chemical compounds, NIST plasma or biological matrix) – Extra information is available for NMR spectra like the optional isotopic labelling – Information about sample preparation will be available in a future planned release
*Liquid chromatography*	Only fullscan and fragmentation LC spectra (LC–MS / LC–MS/MS) “chromatography” sheet	Informs users about chromatography data (column brand, type, name, length, diameter, flow rate, injection volume, gradient…) – The column characteristics can be used as filter in Web service
*Gas chromatography*	Only full scan GC spectra (GC–MS) “chromatography” sheet	Informs users about chromatography data (column brand, type, name, length, diameter, …) – The column characteristics can be used as filter in Web service
*Ion chromatography*	Only fullscan IC spectra (IC-MS) “chromatography” sheet	Informs users about chromatography data (column brand, type, name, length, diameter, …)
*Ionization method*	All mass spectra “MS_analyzer” sheet “GCMS_analyzer” sheet for LC and IC spectra	Informs users about instrument characteristics and settings for the acquisition
*Ion analyzer*	All mass spectra “MS_analyzer” sheet for LC and IC spectra “GCMS_analyzer” sheet for LC and IC spectra	Informs users about instrument characteristics and settings for the acquisition
*NMR instrument* *NMR processing software*	For all NMR spectra “NMR_analyzer” sheet	Informs users about instrument and software characteristics and settings for the acquisition and processing parameters
*“Other” metadata*	For all spectra “Other” sheet	Informs users about the spectrum’s authors, ownership, raw file, …

### Data quality and curation

#### Standardized operating procedures to ensure data quality

Different standard operating procedures (SOPs) provide comprehensive information about the data that can be stored in PeakForest, allowing users to achieve high-quality data levels in local PeakForest databases. All SOPs and publication references are publicly and freely-available on the PeakForest Web portal peakforest.org. To illustrate Web interfaces and MetaboHUB SOPs application, a PeakForest demonstrator is available at https://demo.peakforest.org. This instance contains a dataset of 96 chemical compounds selected for their biological interest and their relatively-easy identification in biological matrices or biofluids. The demonstrator also contains a spectra collection acquired on different analytical platforms for a range of analytical conditions by the MetaboHUB consortium, including flow injection analysis (FIA), reversed-phase (C18) chromatography and hydrophilic interaction chromatography (HILIC) coupled to an Exactive or an Orbitrap Fusion (Thermo Fisher Scientific) or an Impact HDII Quadrupole-Time Of Flight (QToF) (Bruker, Daltonics) mass spectrometer; gas chromatography coupled to Accurate Mass QToF 7200 mass spectrometer (Agilent Technologies, Inc); Avance III NMR spectrometers (Bruker, Biospin) with different environments (magnetic field from 500 to 800 MHz for proton frequency and probes at room temperature to cryoprobes).

#### In silico data checking and automatic/manual curation

The PeakForest data model specifies mandatory data and metadata to be included during import phases, with inbuilt routines to limit or identify mistakes and data inconsistency. For example, PeakForest manages chemical compound unicity, based on the InChI/InChIKey set. If a user imports information about a compound already present in the database, new common names are added to existing ones as synonyms but properties such as the accurate mass are not recomputed. If different external identifiers (e.g. ChEBI ID) are provided, the system does not update them but creates a “curation message” associated with the compound. This curation message text will indicate the nature of the conflict and “curator” users will be able to manually update the entry if a correction is required. Automatic data enrichment within in-silico metadata is also possible. For example, imported compound information can be enriched with in-silico generated physico-chemical properties such as LogP, computed by the OChem Web services (Sushko et al., 2011), and the endogenous mammalian status of molecules are determined using the integrated BioSM tool (Hamdalla et al., 2013).

PeakForest is designed to allow manual data curation with a manual scoring system to rank compound names and a star grading system to distinguish different statuses in compound curation (initial compound import to final validation). Curation is not only feasible on compound descriptors but also on spectral data where, for instance, peak assignments may be added, modified or refined by analytical chemists. Authenticated users on a local PeakForest database can submit messages and raise issues on compound or spectral cards. All these messages are grouped in the PeakForest curation message centre; users with a curator role can manage all reported issues and access the edition mode of all compounds, spectral data and metadata. Users can also associate any scientific publications with a particular chemical compound card through the simple and original paper’s digital object identifier (DOI) or its PubMed identifier.

### Browsing and using PeakForest data

#### PeakForest search interfaces

**Fig. 4:**
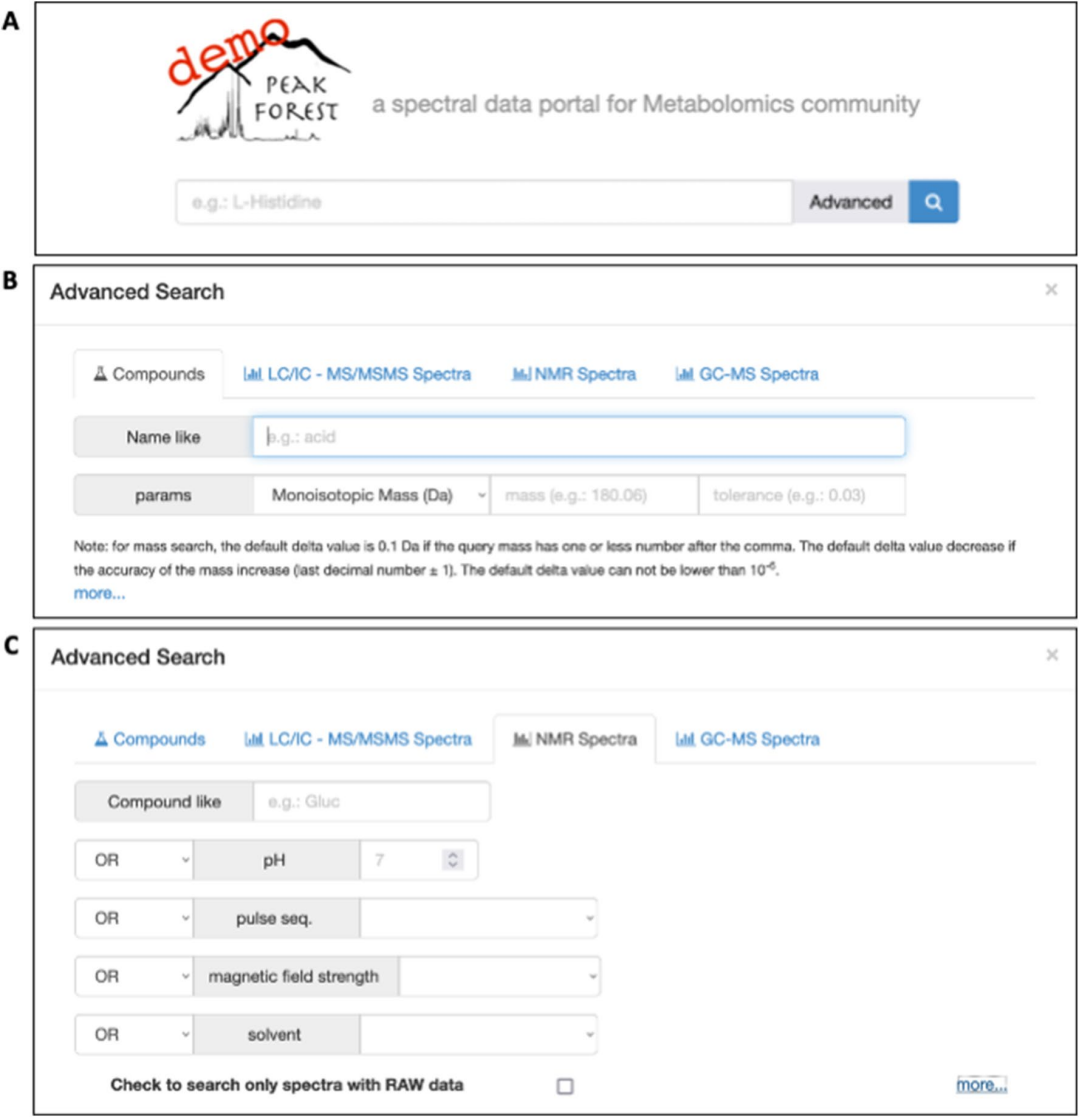
Screenshot of the PeakForest search modules with the “quick search” tool (**A**), and the advanced search tool for compounds (**B**) and for NMR data (**C**)

PeakForest offers specific modules to browse and mine compound and spectral data. The “quick search” Web module uses properties or identifiers (Fig. 4), whereas the “advanced search” module is able to interpret natural language and support advanced keyword queries. A filter system, integrating basic logic gates (‘AND’, ‘OR’) allows users to query the database, and a range of queries are possible depending on spectral type.

#### Exporting data from PeakForest

Since sharing and opening data are as important as building and organizing individual laboratory data and metadata, PeakForest proposes two ways to export local collections into current metabolomics standards. This functionality guarantees the interoperability of PeakForest data collections with common bioinformatics tools, as well as the export into international metabolomics databases (MassBank, MONA, HMDB, GNPS, …) and repositories (MetaboLights, Metabolomics Workbench, …). All compounds and their properties can be exported as a “comma separated value” formatted file, and individual. compound cards can also be exported manually as a SDF (Dalby et al., 1992) formatted file. Spectra are exportable in the MassBank text format and/or specific open formats adapted to different measurement techniques (e.g. nmrML (Schober et al., 2018) for 1D-NMR spectra and MSP for mass spectra) easily linked to common softwares and tools including MS-DIAL (Lai et al., 2018) and GCMS NIST MSsearch.^12^

#### Programmatic interface

The RESTful Web services associated with each local PeakForest infrastructure provide direct access to the complete local data collection. A set of methods are designed to give simple access to compound or spectral cards by exact or partial matching and return information in JSON formatted files. A range of options is also available to facilitate queries including searching for a specific mass-to-charge ratio (m/z) or chemical shift. This Web services layer allows bioinformatics programmers to easily and quickly integrate an existing PeakForest instance as a dynamic data resource with secure access enabled by an authentication token system. PeakForest-compatible tools are currently available as part of the Galaxy project Toolshed^13^ and are hosted on the Workflow4Metabolomics platform.^14^ The complete REST API documentation is available for each local PeakForest database and a generic version is available on the peakforest.org portal. The PeakForest team is committed to develop this API based on queries and contributions submitted via the GitHub repository.

#### PeakForest for spectral data collection of metabolites absent from the chemical library

**Fig. 5:**
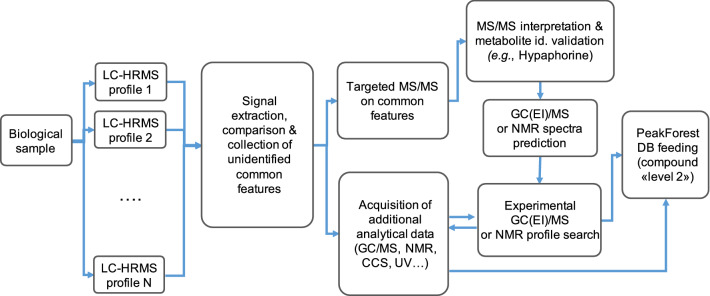
Example of an inter-platform workflow strategy used to generate LC-HRMS/MS, NMR and other orthogonal data related to unknown metabolites in biological matrices, and subsequent PeakForest database enrichment with biological compounds identified with a confidence “MSI level 2”

PeakForest offers the possibility to integrate and share physico-chemical characteristics of not fully elucidated compounds seen recurrently in biological matrices of interest. The identification of these not fully elucidated compounds is facilitated by the aggregation of convergent data from different techniques and analytical platforms (Fig. 5). As a test case, a common and non-assigned signal at m/z *247.1441* that is recurrently detected following LC-HRMS analysis of NIST plasma in positive mode of ionization on two different instruments, an Orbitrap Tribrid Fusion (Thermo Fisher Scientific) and a Q-ToF Impact II (Bruker, Daltonics), led us to further investigations. Additional HRMS/MS acquisitions performed on both instruments allowed to conclude to the presence of a metabolite annotated as hypaphorine in this sample and to list its characteristics (RT, HRMS, MS/MS, etc.) in PeakForest retrieved from each analytical system. With the referencing of these analytical data in PeakForest, hypaphorine (or lenticin) proved to be also detected in human urine (Garcia‐Aloy et al., 2020) and could be annotated with a confidence “level 2” with spectra stored in the MoNA public database.^15^ Additionally, signals obtained with NMR spectra prediction of hypaphorine could be searched for in the experimental NMR profiles of the same biological sample (NIST plasma) in order to confirm its identity (confidence level 2) and enrich the NMR set of data of biological compound in PeakForest. Eventually, when available, the authentic standard may be purchased, analysed and reported in PeakForest, to reach “level 1” annotation.

## Discussion

Improving confidence in the outputs of data annotation and metabolite identification in biological samples is a priority for the metabolomic field (Wishart et al., 2022). Here we present the first comprehensive web-based infrastructure which can simultaneously handle MS and NMR data, and is designed to organize spectral data for metabolome annotation. PeakForest is able to capture and store experimental spectral data (peak lists) as well as associated annotations, and offers the possibility to curate information over time, to be browsed and reused in data treatment and, ultimately, shared across laboratories. PeakForest has the added value of providing a structured system with onsite data centralization and security for small laboratories with limited IT support.

Over the last 15 years, a number of database structures and tools have been proposed to generate local data repositories for either MS or NMR spectral collections, promoting accuracy in metabolite identification (Ferry-Dumazet et al., 2011; Horai et al., 2010; Palmer et al., 2018). PeakForest builds on the principles of these tools, and incorporates the latest developments in web frameworks and applications to provide a unique data model compatible with multiple research questions, depending on laboratory focus and/or instruments. In addition to storing metabolites/spectral metadata, our data model can describe information on sample preparation, biological species identifiers and biological matrices, providing a complete compound profile for future use. For example, a dedicated PeakForest database can be implemented for a particular disease or class of biological species, based on concepts of “sample type-specific databases”. Indeed it has been shown that the use of a restricted search domain increases the precision of annotations (Reisdorph et al., 2019). A key innovation in PeakForest is the capacity to support both MS and NMR spectral data for a multitude of low- and high-resolution configurations and dimensions (i.e., 1D and 2D NMR, MS and MS^2^ data), chromatographic retention times, and seeking to integrate additional structural information or others molecular descriptors, such as ultraviolet–visible spectra and ion mobility mass spectrometric data (collision cross sections, CCS) in the near future. Furthermore, recent studies have called for combined mass and NMR analyses of biological samples in order to improve metabolome annotation and enhance metabolome coverage (Marshall & Powers, 2017). This is particularly relevant for the identification of carbohydrates and their derivatives, which are poorly-detected in LC-HRMS but fully-characterized by GC-HRMS and quantified with NMR (Comte et al., 2021). By centralizing a large diversity of metabolites and profiles, PeakForest proposes a well-adapted database for studies based on a multi-platform untargeted strategy.

Studies in metabolomics are increasingly using high-throughput screening technologies and large sample batches; these approaches generate “big data” and bring new challenges regarding data management and security. The key to efficient data management involves setting up appropriate data stewardship during production, treatment, mining and knowledge dissemination. In this context, global guidelines are now available to help specific metabolomics communities to enhance their data usage and new knowledge creation (Savoi et al., 2021). However, applying these recommendations can be complex and often requires changes in traditional work practices, new skills and/or training (Griffin et al., 2018). PeakForest complies with “best practice” data management guidelines via SOPs which comprehensively address the data production workflow, from sample collection and preparation, chemotype analysis and data treatment to metabolite annotation. It is designed as a complementary component of effective information systems, and can be used in conjunction with laboratory information management systems (LIMS) which provide high traceability for sample data and metadata (Hunter et al., 2017). PeakForest is also compatible with modern data analysis workflows-based platforms which generate metadata reproducibility for data analyses (Giacomoni et al., 2015; Guitton et al., 2017; Huan et al., 2017; Pang et al., 2021; Tautenhahn et al., 2012; Xia & Wishart, 2011).

In line with the open-science movement, the metabolomics community has made significant efforts to align their standards with FAIR (Findable, Accessible, Interoperable, and Reusable) data principles through the creation and the use of open data formats and online resources (Mendez et al., 2019). PeakForest adopts FAIR practices (Johnson & Lange, 2015; Wilkinson et al., 2016) in order to ensure that data at the laboratory level is consistent with, and can be used by, the international metabolomics community. PeakForest uses standardized vocabulary, including accurate and relevant attributes. Metabolomics annotation recommendations have been implemented, such as assigning cross reference ID on metabolites and making sharable reference spectra (Kind et al., 2018; Redestig et al., 2010), and use of unambiguous and persistent identifiers for metabolites and LC–MS data (Wohlgemuth et al., 2016) and will facilitate the future integration of such data in data contextualization tools (Cottret et al., 2018; Delmas et al., 2021). Interoperability with data analysis and data mining tools is ensured by open communication protocols (REST), and data export functions with conversion into common open standards formats ensure interoperability with academic databases and open repositories in the metabolomics community. Overall, the PeakForest framework is designed to encourage greater openness of laboratory databases, facilitating integration into public databases and knowledge sharing between laboratories, and ultimately promoting effective metabolomics research (fewer redundancies across laboratories, faster annotation of unknowns).

PeakForest has been extensively beta-tested by the members of the French metabolomics community. It is fully-operational (available at https://peakforest.org/), and already currently used in research laboratories in France and abroad. In order to minimize server storage of unnecessary data, PeakForest is designed for metabolite annotation report data rather than storage of raw spectral data. Technical installation of PeakForest in any laboratory information system requires personnel with computing or bioinformatics skills, but once installed, the interface is user-friendly and the infrastructure is adapted for use by MS and NMR scientists with no specific computer skills. Of course, a minimum number of compounds and spectra need to be imported by users in order to make local PeakForest databases fully-functional. In addition, PeakForest does not include data clean-up or extraction tools per se and requires metadata to be manually entered in the database; users supply completely-cleaned data following SOPs edited by the PeakForest team, and PeakForest tutorials are available to assist in this step. Data and metadata within PeakForest can be changed (curated) with ease; traceability of requested modifications is ensured via curation modules. This feature is ideally-suited to local data facilities under the supervision of a data manager, instrumental experts or associated scientists, but is less easy to deploy in larger-scale public repositories as it requires centralised and standardized highly-reactive error detection and correction processes.

As with all databases, initial construction of the relevant database and spectral collection in line with laboratory needs will take some time. Deployment of PeakForest is an iterative process, and initial investment in the database will yield increasing returns in terms of accuracy and efficiency as the spectral collections are incremented. At present a number of basic peak-matching tools are available as add-ons via the Galaxy toolshed, but additional developments are needed to enhance the MS–MS and NMR-2D matching capabilities, such as interoperability with MS-Dial (Tsugawa et al., 2015). For laboratories with access to bioinformatics support, integration of bespoke tools to the PeakForest infrastructure can contribute the optimization of local data pipelines analysis and metabolite annotation. This adaptability of PeakForest system offers multiple perspectives, and the opportunity to meet the evolving needs of the metabolomics research community.

## Conclusion

This paper describes a novel digital infrastructure for the development of “new generation”, structured and interoperable databases. PeakForest is intended to overcome a technical bottleneck, namely large-scale collaborative spectral data annotation and metabolite identification for metabolomics laboratories with multiple instruments. The PeakForest database is a user-friendly data management solution, which can be used to build metabolite and spectral collections, and has in-built modules which allow users to curate and mine annotation mass and NMR data via Web interfaces and external tools. By generating an ecosystem of interoperable databases, PeakForest represents a significant advance for promoting open science in the field of metabolomics, maintaining and promoting good practice and scientific rigour, as well as increasing the shareability and findability of data. The PeakForest digital infrastructure is associated with a public portal proposing technical guides, tutorials and SOPs. Integration of PeakForest in the Galaxy workflow environment facilitates the adoption and personalization of local databases by bioinformatics developers associated with metabolomics facilities, and the emergence of new tools for the scientific community based on a common standard. Finally, PeakForest also offers opportunities for future alignments with additional existing biological and chemical ontologies.

## Supplementary Information

Below is the link to the electronic supplementary material.

Supplementary file1 PeakForest framework, technical specifications, API detail and user rights (DOCX 207 kb)

Supplementary file2 PeakForest template file for compounds import. (XLSX 49 kb)

Supplementary file3 PeakForest template file for LC-MS type spectra import in the case of a chemical standard. (XLSX 1078 kb)

Supplementary file4 PeakForest template file for GC-MS type spectra import in the case of a chemical compound mix. (XLSX 1078 kb)

Supplementary file5 PeakForest template file for LC-MSMS type spectra import in the case of an analytical matrix (urine from *homo sapiens*). (XLSX 1291 kb)

Supplementary file6 PeakForest template file for NMR (1D or 2D) type spectra import in the case of a standardized matrix. (XLSX 1073 kb)

Supplementary file7 Compound and Spectral data origins (DOCX 13 kb)

Supplementary file8 Author contributions (DOCX 32 kb)

## Data Availability

This paper does not present data but a digital solution. A PeakForest demonstrator is available at https://demo.peakforest.org. This instance contains an example dataset of 96 chemical compounds and 400 spectra.

## Footnotes

1 https://massbank.eu.

2 https://mona.fiehnlab.ucdavis.edu.

3 https://peakforest.org/local_install.

4 https://peakforest.org/howto.

5 https://hub.docker.com/u/metabohub.

6 https://cecill.info/licences/Licence_CeCILL_V2.1-en.html.

7 https://github.com/peakforest.

8 https://www.app.asso.fr/en.

9 http://openbabel.org/.

10 https://peakforest.org/descriptors.

11 http://www.mssj.jp/english/about/pdf/MassBank_manual-en.pdf.

12 https://www.nist.gov/system/files/documents/srd/nistms.pdf.

13 https://toolshed.g2.bx.psu.edu/.

14 https://workflow4metabolomics.usegalaxy.fr/.

15 https://mona.fiehnlab.ucdavis.edu/spectra/display/VF-NPL-QEHF001173.

## Software availability

Demo: https://demo.peakforest.org/webapp/; Project Web portal: https://peakforest.org/; Code repository (Major releases): https://github.com/peakforest; Docker files: https://hub.docker.com/u/metabohub.
