# Supplementary material for: PeakForest: a multi-platform digital infrastructure for interoperable metabolite spectral data and metadata management
Source: Metabolomics. 2022 Jun 14;18(6):40. doi: 10.1007/s11306-022-01899-3 (PMC9197906; doi:10.1007/s11306-022-01899-3)
Supplement: Supplementary file 1 — Supplementary file1 PeakForest framework, technical specifications, API detail and user rights (DOCX 207 kb) [file 11306_2022_1899_MOESM1_ESM.docx]

**Online Resource 1: PeakForest framework, technical specifications, API detail and user rights**

PeakForest is organized as several complementary API projects (Figure ESM_1)

**
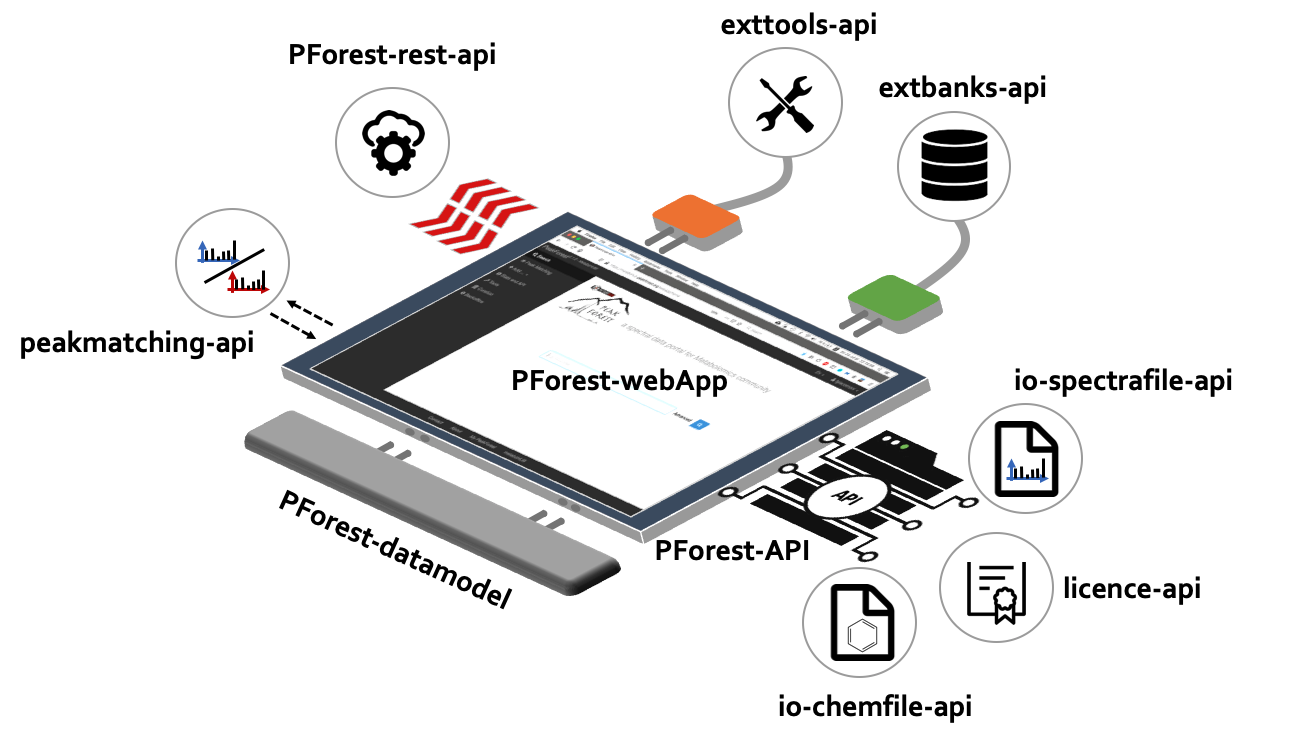
**

Figure ESM_1: PeakForest general structure and API components

PeakForest is built on the ***Spring MVC framework^[[1]](#footnote-1)^*** for the back-end. This framework confers robust and up-to-date functionalities for user authentication, rights management and password encryption with Spring security and at the global application security level, prevents cross-site scripting (XSS) attacks on PeakForest Web pages with Jsoup^[[2]](#footnote-2)^. The application is based on the Hibernate java Object-relational mapping (ORM) framework, designed to avoid structured query language (SQL) injection attacks and coupled with a ***MySQL^[[3]](#footnote-3)^*** relational database. Java dependencies are managed using Maven. The PeakForest front-end uses the modern and ergonomic ***Twitter-Bootstrap^[[4]](#footnote-4)^*** template associated with the ***Font Awesome icons^[[5]](#footnote-5)^*** library and Bootstrap and jQuery add-ons in order to improve user experience. PeakForest leverages on adapted Javascript-based libraries: ***HighCharts^[[6]](#footnote-6)^*** for mass spectra and ***Handsontable^[[7]](#footnote-7)^*** for Excel-like sheet in Web-pages, ***NMRPro^[[8]](#footnote-8)^*** for 1D and 2D NMR spectra, ***GLmol^[[9]](#footnote-9)^*** and ***Jmol^[[10]](#footnote-10)^*** to display 3D compounds representations, and ***MetExploreViz^[[11]](#footnote-11)^*** (Chazalviel et al., 2018) for metabolite mapping in metabolic networks. The system includes the open-source component ***OpenBabel^[[12]](#footnote-12)^*** (O’Boyle et al., 2011) to compute and confirm properties associated with chemical compounds and ***Discovery^[[13]](#footnote-13)^***, a novel javascript library to infer PeakForest data with metabolomics Web-linked resources.

***PeakForest API details***

- Data-Model: defines the PeakForest data-model and the MySQL database schema using hibernate annotations. The data-model entities contain annotations for JSON dumping.
- ExternalBanks-API: used to centralize third-party Web services clients to online resources like PubChem, MetExplore, ChEBI, CTS, … This application is designed to be used as a standalone software to query these Web services.
- ExternalTools-API: used to call third-party software on the local computer. To import chemical compounds, PeakForest depends on:
- OpenBabel (<https://jcheminf.biomedcentral.com/articles/10.1186/1758-2946-3-33> ) to compute chemical properties from the compound’s InChI. This software is also used to generate the compounds’ MOL files and SVG images. The installation of this software can be tricky, so please refer to the documentation.
- NMR Reader is a java library used to extract metadata from NMR brucker files.
- BioSM is a java library to determine if a compound is a mammalian endogenous metabolite, using its SMILES format.
- phantomJS is a back-end tool able to generate images from Web-pages. We use it to create thumbnails of NMR spectra representation.
- NMRPro - Server: (<https://github.com/ahmohamed/nmrpro> ) is a python software able to compute data to display 1D and 2D NMR spectra in the front-end.
- IO-SpectraFile-API: used to import Spectra entities from XLSM template files and map them into PeakForest compliant ones.
- IO-ChemicalFile-API: used to import Chemical entities from XLS template files and map them into PeakForest compliant ones.
- Other small APIs were developed as light toolboxes (PeakForest-Utils, PeakMatching-API, Licence-API) or to manage reference files through maven dependencies in other PeakForest projects (PeakForest-Templates-Files, PeakForest-REST-OpenAPI-File...)
- PeakForest-API: the main core API that contains DAO (Data access object), Services classes to read and write entities in the database. This API is an orchestrator for all smaller ones.

***User rights***

Roles are organized as four levels: anonymous, validated user, curator and administrator. An **anonymous user** is a user with no pre-existing account (or not logged on). This level allows access to “search” pages, compound/spectra cards, PeakForest instance statistics and external plugged tools. The **validated user** has an activated PeakForest account, and can also add compound/spectra cards and write curation messages. The **curator** role allows users to add, delete and edit compound/spectra cards and to fix curation messages. The **administrator** role offers the possibility to manage users (user validation or deletion and role settings) and the server (status, database tools, license and analytics).

1. https://spring.io/projects/spring-framework/ [↑](#footnote-ref-1)
2. https://jsoup.org/cookbook/cleaning- [↑](#footnote-ref-2)
3. http://www.mysql.com/ [↑](#footnote-ref-3)
4. https://startbootstrap.com/theme/ [↑](#footnote-ref-4)
5. https://fontawesome.com/ [↑](#footnote-ref-5)
6. https://www.highcharts.com/ [↑](#footnote-ref-6)
7. https://handsontable.com/ [↑](#footnote-ref-7)
8. https://github.com/ahmohamed/nmrpro [↑](#footnote-ref-8)
9. http://Webglmol.osdn.jp/ [↑](#footnote-ref-9)
10. https://sourceforge.net/projects/jmol/ [↑](#footnote-ref-10)
11. https://metexplore.toulouse.inrae.fr/metexploreViz/doc/documentation.php [↑](#footnote-ref-11)
12. http://openbabel.org/ [↑](#footnote-ref-12)
13. https://github.com/p2m2/Discovery [↑](#footnote-ref-13)
