# Supplementary material for: PeakForest: a multi-platform digital infrastructure for interoperable metabolite spectral data and metadata management
Source: Metabolomics. 2022 Jun 14;18(6):40. doi: 10.1007/s11306-022-01899-3 (PMC9197906; doi:10.1007/s11306-022-01899-3)
Supplement: Supplementary file 7 — Supplementary file7 Compound and Spectral data origins (DOCX 13 kb) [file 11306_2022_1899_MOESM7_ESM.docx]

**Online Resource 7: Compound and Spectral data origins:**

| **Data** | **Origins** |
| --- | --- |
| Compounds InChI | Added by PeakForest’s users |
| Compound InChIKey | Set by PeakForest’s users, cross-checked from InChIKey using OpenBabel algorithms |
| Compound common names, IUPAC name, external databases IDs, CAS numbers | Set by PeakForest’s users. Can be copied from the CTS Web service if compounds are registered from Web-GUI. |
| Compounds bibliographical references | Set by PeakForeset’s users. Publications’ data are fetched from the PubMed Web service using publications’ PubMed IDs or DOI. |
| Compound monoisotopic and average masses, default MOL and SVG representations, canonical SMILES | Computed thanks OpenBabel from compounds’ InChI |
| Manual curated compounds MOL representation | Fetched from CACTUS Web service using compounds’ InChIKey as identifier |
| Compound numbered structures | Created and uploaded into PeakForest by the users |
| Compound logP value | Computed thanks OChem’s Web service from compounds’ SMILES |
| Compound MetaboLigths references | Collected on the fly from FORUM’s MetaboLights triplestore using Discovery’s tools (Publication submitted). |
| Spectra Metadata and PeakList | Registered by PeakForest’s users using either the Web-GUI or the XLSM template files |
| NMR spectra representation | Computed from spectra’s Brucker file, previously uploaded by PeakForest users. Displayed thanks to NmrPro viewer. |
| Mass spectra splash ID | Computed from mass spectra’s peak list using fiehnlab’s tool (java library) |
| NMR spectra basic data | Extracted from spectra’s bruker files using “nmrRead tool” by Daniel Jacob (java library) |
