# Supplementary material for: PeakForest: a multi-platform digital infrastructure for interoperable metabolite spectral data and metadata management
Source: Metabolomics. 2022 Jun 14;18(6):40. doi: 10.1007/s11306-022-01899-3 (PMC9197906; doi:10.1007/s11306-022-01899-3)
Supplement: Supplementary file 8 — Supplementary file8 Author contributions (DOCX 32 kb) [file 11306_2022_1899_MOESM8_ESM.docx]

**Online Resource 8: Author contributions (based on the CRediT – Contributor Roles Taxonomy)**

| Author | Concept | Data curation | Formal Analysis | Funding acquisition | Investigation | Methodo. | Project administr. | Resources | Software | Supervision | Validation | Visual. | Writing – original draft | Writing – review & editing |
| --- | --- | --- | --- | --- | --- | --- | --- | --- | --- | --- | --- | --- | --- | --- |
| Nils Paulhe (NP) | x |  |  |  |  | x |  | x | x |  |  | x | x | x |
| Cécile Canlet (CC) | x | x | x |  | x | x |  |  |  |  | x | x | x | x |
| Annelaure Damont (Ada) | x | x | x |  | x | x |  |  |  |  | x | x | x | x |
| Lindsay Peyriga (LP) | x | x | x | x | x | x |  |  |  |  | x |  |  | x |
| Stephanie Durand (SDu) | x | x | x |  | x | x |  |  |  |  | x | x |  | x |
| Catherine Deborde (CDe) | x | x | x |  | x | x |  |  |  |  | x |  |  | x |
| Sandra Alves (SA) | x | x |  |  | x | x |  |  |  |  | x |  |  |  |
| Stephane Bernillon (SB) | x | x | x |  | x | x |  |  |  |  | x |  |  |  |
| Thierry Berton (TB) |  | x | x |  | x |  |  |  |  |  | x |  |  |  |
| Raphael Bir (RB) |  | x | x |  | x |  |  |  |  |  | x |  |  |  |
| Alyssa Bouville (AB) | x | x | x |  | x | x |  |  |  |  | x |  |  |  |
| Edern Cahoreau (EC) | x | x | x |  | x | x |  |  |  |  | x |  |  | x |
| Delphine Centeno (DC) | x | x | x |  | x | x |  |  |  |  | x |  |  | x |
| Robin Costantino (RC) |  | x | x |  | x |  |  |  |  |  | x |  |  |  |
| Laurent Debrauwer (LD) | x |  |  | x |  | x |  |  |  |  |  |  |  | x |
| Alexis Delabriere (AD) | x |  |  |  |  |  |  |  | x |  |  |  |  |  |
| Christophe Duperier (CD) | x |  |  |  |  | x |  | x |  |  | x |  |  |  |
| Sylvain Emery (SE) |  | x | x |  | x |  |  |  |  |  | x |  |  |  |
| Amelie Flandin (AF) |  | x | x |  | x |  |  |  |  |  | x |  |  |  |
| Ulli Hohenester (UH) | x | x | x |  | x | x |  |  |  |  | x |  |  |  |
| Daniel Jacob (DJ) | x | x |  |  |  | x |  |  | x |  | x |  |  | x |
| Charlotte Joly (CJol) | x | x | x |  | x | x |  |  |  |  | x |  |  | x |
| Cyril Jousse (CJou) | x | x |  |  | x | x |  |  |  |  | x |  |  |  |
| Marie Lagree (ML) | x | x | x |  | x | x |  |  |  |  | x |  |  |  |
| Nadia Lamari (NL) |  | x | x |  | x |  |  |  |  |  | x |  |  |  |
| Marie Lefebvre (MLe) | x |  |  |  |  | x |  |  | x |  | x |  |  |  |
| Claire Lopez-Piffet (CLP) | x | x | x |  | x | x |  |  |  |  | x |  |  |  |
|  |  |  |  |  |  |  |  |  |  |  |  |  |  |  |
| Author | Concept | Data curation | Formal Analysis | Funding acquisition | Investigation | Methodo. | Project administr. | Resources | Software | Supervision | Validation | Visual. | Writing – original draft | Writing – review & editing |
| Bernard Lyan (BL) | x | x | x |  | x | x |  |  |  |  | x |  |  |  |
| Mickael Maucourt (MM) |  | x | x |  | x |  |  |  |  |  | x |  |  |  |
| Carole Migné (CM) | x | x | x |  | x | x |  |  |  |  | x |  |  |  |
| M.F Olivier (MFO) | x | x | x |  | x | x |  |  |  |  | x |  |  |  |
| Estelle Rathahao-Paris (EPR) | x | x | x |  | x | x |  |  |  |  | x |  |  |  |
| Pierre Petriacq (PP) | x | x |  | x |  |  |  |  |  |  | x |  |  |  |
| Julie Pinelli (JP) |  | x | x |  | x |  |  |  |  |  | x |  |  |  |
| Léa Roch (LR) |  | x | x |  | x |  |  |  |  |  | x |  |  |  |
| Pierrick Roger (PR) | x | x |  |  |  |  |  |  | x |  | x |  |  |  |
| Simon Roques (SR) |  | x | x |  | x |  |  |  |  |  | x |  |  |  |
| Jean-Claude Tabet (JCT) | x | x |  |  |  | x |  |  |  |  |  |  | x | x |
| M. Tremblay-Franco (MTF) | x |  |  |  |  | x |  |  | x |  | x |  |  |  |
| Mounir Traïkia (MT) | x | x | x |  | x | x |  |  |  |  | x |  |  |  |
| Anna Warnet (AW) | x | x | x |  | x | x |  |  |  |  | x |  |  |  |
| Vanessa Zhendre (VZ) |  | x | x |  | x |  |  |  |  |  | x |  |  |  |
| Dominique Rolin (DR) |  |  |  | x |  |  | x |  |  | x |  |  |  | x |
| Fabien Jourdan (FJ) | x |  |  | x |  |  |  |  |  | x |  |  | x | x |
| Etienne Thevenot (ET) | x |  |  |  |  | x |  |  |  | x |  |  |  | x |
| Annick Moing (AM) | x |  |  |  |  | x |  |  |  |  | x |  |  | x |
| Emilien Jamin (EJ) | x | x | x |  |  | x |  |  |  |  | x |  |  | x |
| Francois Fenaille (FF) | x | x |  | x |  | x |  |  |  |  | x |  | x | x |
| Christophe Junot (CJ) | x |  |  | x |  |  | x |  |  | x |  |  | x | x |
| Estelle Pujos-Guillot (EPG) | x | x |  | x |  |  | x |  |  | x | x | x | x | x |
| Franck Giacomoni (FG) | x | x | x |  | x | x | x | x | x | x | x | x | x | x |

*Conceptualization, Data curation, Formal Analysis, Funding acquisition, Investigation, Methodology, Project administration, Resources, Software, Supervision, Validation, Visualization, Writing – original draft, Writing – review & editing are the 14 contributor roles and definitions are available at https://casrai.org/credit/)*
